# Criterion validity of a single-item measure of fear avoidance behavior following mild traumatic brain injury

**DOI:** 10.1186/s12883-024-03861-3

**Published:** 2024-09-28

**Authors:** Shahrazad Amin, Ana Mikolic, Noah D. Silverberg

**Affiliations:** 1 Department of Psychology, University of British Columbia, Vancouver, British Columbia Canada; 2 Department of Psychology, Simon Fraser University, Burnaby, British Columbia Canada; 3 Rehabilitation Research Program at GF Strong Rehab Centre, Centre for Aging SMART, Vancouver, British Columbia Canada

**Keywords:** Fear avoidance, mTBI, Outcome measures, Psychometrics

## Abstract

Maladaptive coping such as fear avoidance behavior can prolong recovery from mild traumatic brain injury (mTBI). Routine assessment of fear avoidance may improve management of mTBI. This study aimed to validate a single-item measure of fear avoidance to make its assessment more pragmatic. The present study is a secondary analysis of a clinical trial that involved adults with persistent post-concussion symptoms (*N* = 90, 63% female). Participants completed the single-item fear avoidance rating, a validated legacy measure of fear avoidance (Fear Avoidance Behavior after Traumatic Brain Injury; FAB-TBI), and measures of anxiety (General Anxiety Disorder-7), depression (Patient Health Questionnaire-9), post-concussion symptoms (Rivermead Postconcussion Symptoms Questionnaire), and disability (World Health Organization Disability Assessment Schedule 12.0). Questionnaires were completed twice, at baseline (mean 18.1 weeks post injury) and again 12–16 weeks later following study-delivered rehabilitation in addition to usual care. We analyzed the associations (Spearman’s correlations) and agreement (weighted Kappa) between the single-item and FAB-TBI at baseline, posttreatment, individual FAB-TBI item scores, and the change in scores between baseline and posttreatment. In addition, we examined correlations between the single-item fear avoidance measure and related constructs, including anxiety, depression, post-concussion symptoms, and disability. The single-item fear avoidance measure correlated strongly with the FAB-TBI both at baseline and following treatment (*ρ* = 0.63 − 0.67, *p* < .001), and moderately with FAB-TBI item scores (*ρ* = 0.4 − 0.6). The correlation between the change in these scores from baseline to posttreatment was moderate (*ρ* = 0.45, *p* < .001). Agreement between the single-item fear avoidance measure and discretized FAB-TBI scores was moderate (κ = 0.45 − 0.51). Before and after treatment, the single-item fear avoidance measure correlated moderately with anxiety (*ρ* = 0.34), depression (*ρ* = 0.43), post-concussion symptoms (*ρ* = 0.50), and disability (*ρ* = 0.43). The FAB-TBI was more strongly correlated with these measures (*ρ* = 0.53 − 0.73). In summary, the present study supports the criterion validity of the single-item fear avoidance measure. This measure may be a useful screening and monitoring tool for patients with mTBI but is not a substitute for the FAB-TBI questionnaire.

**Supplementary Information:**

The online version contains supplementary material available at 10.1186/s12883-024-03861-3.

## Introduction

Every year, 50–60 million people sustain a traumatic brain injury worldwide and 80–90% of TBIs are classified as mild [1]. At least 20% of adults with mild TBI experience persistent post-concussion symptoms, such as headaches, memory problems, and fatigue [2]. Maladaptive coping with post-concussion symptoms can hinder recovery from mild TBI. Perhaps the best studied maladaptive coping style after mild TBI to date is fear avoidance behavior. Fear avoidance refers to avoiding or escaping from activities and situations that the injured person expects might aggravate their symptoms or result in other harms (e.g., re-injury, social embarrassment). Prolonged fear avoidance is associated with worse post-concussion symptoms and disability [3–6], heightened sensitivity to symptom provocation [7, 8], and lower rates of return to work [9]. Targeting fear avoidance psychologically-informed rehabilitation may improve mild TBI outcomes [10].

Most prior studies have measured fear avoidance behavior after mild TBI with the 16-item Fear Avoidance Behavior after Traumatic Brain Injury Questionnaire (FAB-TBI) [11]. This scale was adapted from existing measures of fear avoidance in the chronic pain literature. The FAB-TBI has high internal consistency and acceptable unidimensionality [11], and clinical reference values are available [3]. With 16 items, the FAB-TBI is somewhat lengthy, which can be prohibitive for regular use in clinical settings and in research studies where fear avoidance may be relevant, but is not of primary interest.

An ultra-brief fear avoidance scale would allow for more widespread measurement of this important construct. Single-item measures are useful for screening (e.g., at clinic intake, to inform whether more in-depth assessment is needed, such as with the FAB-TBI), serial monitoring (e.g., weekly during a course of treatment), for research involving ecological momentary assessment (which involve intensive repeated measurements) [12], and other circumstances involving time constraints. The benefits of single-item measures include minimal administration time, reduced participant burden, and greater content validity [13]. Single-item measures of psychological constructs can be reliable and valid, such as measures for depression and anxiety [12–14]. However, there are generally tradeoffs of weaker psychometric properties compared to full length scales [13]. Under the classical test theory, adding items to a scale improves reliability and the ratio of true score variance to error variance [15]. Thus, it is more difficult to establish reliability and measurement error in single-item measures according to the principles of this theory. The present study aimed to evaluate a single-item fear avoidance measure.

## Aims

This study aims to investigate the criterion validity of a brief, single-item fear avoidance measure following mild TBI. We report the association and agreement between the single-item fear avoidance measure and the legacy FAB-TBI scale. We further examined associations between the single-item fear avoidance measure and clinical outcomes that are correlated with fear avoidance as measured by the FAB-TBI [11], including depression, anxiety, post-concussion symptoms, and disability.

## Methods

### Study population

This is a secondary analysis of a randomized controlled trial investigating the feasibility of two behavioral interventions following concussion [10]. The sample was comprised of *N* = 90 adults (< 70 years old) who were recruited from two public sector concussion clinics in the Greater Vancouver area, British Columbia, Canada: GF Strong Adult Concussion Service and the Fraser Health Concussion Clinic. These interdisciplinary clinics offer a group-based education session about mTBI symptom management, facilitated by and occupational therapist, and then additional visits with an occupational therapist, physical therapist, neuropsychologist, and/or physician, as needed. Research assistants attended the education sessions to introduce the parent study and invite patients to consent to be contacted for eligibility screening. In addition to usual care (described above), participants in the parent research study received 8 manualized individual videoconference sessions with an occupational therapist and psychology provider dyad. The content of those sessions differed based on random assignment (graded exposure to avoided activities and situations vs. operant conditioning-based pacing strategies) [10].

Eligible participants had persistent post-concussion symptoms and high avoidance and/or endurance behavior [10]. Persistent symptoms were defined as endorsing 3 or more moderate-severe symptoms on the Rivermead Postconcussion Symptom Questionnaire [16] at the time of eligibility screening. Participants completed questionnaires twice: once at clinic intake (M = 18.2 weeks post injury) and again 12–16 weeks later (M = 32.2 weeks post injury, *n* = 82) following treatment. Information about participant demographics and injury mechanism were collected at baseline. Ninety participants completed both the single-item fear avoidance measure and FAB-TBI at baseline/pre-treatment, and 82 of these participants also completed both measures posttreatment. The study was approved by the Clinical Research Ethics Board at the University of British Columbia (H18-02344). Detailed methods for the parent study are reported elsewhere [10].

### Measures

#### Single-item fear avoidance measure

Participants were asked how strongly they agree with the following statement “*I avoid activities that might make my symptoms worse*” over the past week, on a 10-point Likert scale from 1 (*Strongly disagree*) to 10 (*Strongly agree*). The item was developed by an author of the FAB-TBI (NDS) for the purpose of this study.

### Fear avoidance behavior after traumatic brain injury questionnaire (FAB-TBI)

The FAB-TBI is a validated 16-item self-report scale [11]. Items are assessed on a scale of 0 (*Strongly disagree*) to 3 (*Strongly agree*), where high scores indicate greater fear avoidance behavior. It includes items that reflect cogniphobia (e.g., “*I worry that when I have to think or concentrate too hard that I will bring on a headache*”), activity avoidance (e.g., “*I have avoided my usual activities*”), and symptom avoidance (e.g. “*I stop what I am doing when my symptoms start to get worse*”). Raw scores are converted into Rasch scores to convert ordinal scores to a linear scale [11].

### Patient health questionnaire 9 (PHQ-9)

The PHQ-9 is a 9-item self-report measure of depression symptom severity [17]. Each of the items corresponds to one of the criteria that assesses depressive disorders in the Diagnostic and Statistical Manual of Mental Disorders, fourth edition (DSM-IV), where scores are assessed from 0 (*Not at all*) to 3 (*Nearly every day*). The total score is the sum of all the individual items. The PHQ-9 has demonstrated excellent internal reliability (Cronbach’s α = 0.89) and test-retest reliability and is a valid measure of depression severity [17].

### General anxiety questionnaire 7 (GAD-7)

The GAD-7 is a 7-item self-report measure of generalized anxiety [18]. Items are assessed on a four-point rating scale ranging from 0 (*Not at all*) to 4 (*Nearly every day*). The total score of the GAD-7 is the sum of the scores on the 7 items. The GAD-7 has good test-retest reliability, construct validity, and criterion validity, and excellent internal consistency (Cronbach α = 0.92).

### World health organization disability assessment (WHODAS)

World Health Organization Disability Assessment Schedule 2.0 12-item interviewer version assesses functioning across six domains of the International Classification of Disability, Functioning, and Health [19]: cognition, mobility, self-care, interpersonal functioning, life activities, and participation. The WHODAS demonstrates high internal consistency (Cronbach’s α = 0.86) and excellent test-retest reliability in a general population. The WHODAS has also been evaluated in a mild TBI sample, where it showed high internal consistency (Cronbach’s α = 0.92) and adequate construct and concurrent validity [20]. A Rasch transformation to WHODAS scores was applied to enhance psychometric properties in a mTBI sample, using ordinal to interval score conversions [20].

### Rivermead postconcussion symptoms questionnaire (RPQ)

The RPQ is a 16-item self-report scale that assesses symptom severity following mTBI [16]. Participants were asked to compare their symptoms in the past 24 h to prior to their injury on a scale from 0 (*Not experienced at all*) to 4 (*A severe problem*). Some symptoms assessed by the RPQ include headaches, dizziness, nausea, sleep disturbances, fatigue, and concentration problems. The total score is the sum of all the individual items with 1 (“no more of a problem”) recoded as 0.

### Statistical analyses

Information about participant demographics and injury characteristics were described in terms of mean, standard deviation, and frequency. To assess the criterion validity of the single-item fear avoidance measure, we examined its correlations (*ρ*) with the FAB-TBI total score and individual items, as well as its agreement with the FAB-TBI. Weighted Kappa was used to assess agreement between the single-item fear avoidance measure and FAB-TBI at baseline, posttreatment, and the change in scores between baseline and posttreatment [21]. The weighted Kappa analysis involved first converting the continuous (interval-level) FAB-TBI total score to an ordinal scale with 10 levels, to align with the single-item fear avoidance measure. Specifically, we multiplied FAB-TBI scores by 10/48 and rounded the products to whole numbers. Change in scores was calculated by subtracting scores at baseline from the scores posttreatment [(score posttreatment) – (score at baseline)], for both the single-item fear avoidance measure and FAB-TBI.

The convergent validity of the single-item fear avoidance measure was assessed using correlation with related measures, including post-concussion symptoms (RPQ) [16], depression (PHQ-9) [17], anxiety (GAD-7) [18], and disability outcomes (WHODAS) [19]. In addition, we calculated the correlation between change in single-item and FAB-TBI scores before and after treatment. Spearman’s rank correlation coefficient (*ρ*) was used due to the non-normal distribution of single-item avoidance measure scores (Appendix 1a, 1b) and monotonic relationship between single item and other measures. To facilitate comparisons, we report Spearman’s *ρ* for all other correlations. Statistical analyses were conducted using R version 1.4.1103 and SPSS version 27.

## Results

### Descriptive statistics

The current study included *N* = 90 participants at baseline and *n* = 82 participants posttreatment. Of the eight participants that did not complete their assessment posttreatment, two participants (*n* = 2) withdrew from the study and six (*n* = 6) participants did not complete their outcome assessment following treatment. 69% of participants completed the baseline assessment between 2 and 6 months post injury. The majority of included participants were women (63.3%) and White (75.6%). The median age of the sample was 40.5 years (range = 20–65). See Table 1 for full demographic and injury characteristics.

**Table 1:** Participant demographic and clinical characteristics (*N* = 90)

Variable	Value
Age, Mean (SD),	41.59 (11.1)
Sex, n (% female)	57 (63.3%)
Ethnicity, n (%) White Asian Middle Eastern African Indigenous Hispanic	67 (75.6%) 15 (16.5%) 5 (5.5%) 1 (1.1%) 1 (1.1%) 1 (1.1%)
Years Education, M (SD)	15.46 (2.31)
Mechanism of Injury, *n* (%) Moving head Whiplash no contact Moving object	63 (70.0%) 18 (20.0%) 9 (10.0%)
LOC, *n* (%) Yes Suspected No Unknown	11 (12.2%) 10 (11.1%) 62 (68.9%) 5 (5.6%)
PTA, *n* (%) Yes No Unknown	43 (47.8%) 46 (51.1%) 1 (1.1%)
Altered mental status Yes No Unknown	84 (93.3%) 4 (4.4%) 1 (1.1%)

*Note* M = Mean, SD = Standard Deviation, LOC = Loss of consciousness; PTA = post-traumatic amnesia

Descriptive statistics for the full sample are shown in Table 2. In terms of single-item avoidance measure scores at baseline/pre-treatment, the median score was 6 (Q1- Q3: 3–8). Posttreatment, the median score was 3 (Q1- Q3: 1.25-6). The total score of the single-item fear avoidance measure showed minimal floor (7.8%, *n* = 7) and ceiling effects (14.4%, *n* = 13) at baseline. At follow up, a greater percentage demonstrated floor levels (23.3%, *n* = 21), however the ceiling effects were diminished (3.7%, *n* = 3). Frequency distributions for single-item avoidance measure (Appendix 1a, 1b) and the FAB-TBI (Appendix 2a, 2b) are available in the supplementary materials.

**Table 2:** Descriptive statistics of psychological self-report measures

Scale	Baseline		Posttreatment	
	Mean (SD)	Min - Max	Mean (SD)	Min - Max
Single-item fear avoidance	5.8 (2.87)	1–10	3.9 (2.72)	1–10
FAB-TBI Rasch	23.7 (7.23)	6.05–48.0	18.2 (6.89)	0–34.9
GAD-7	9.2 (5.15)	0–21	5.8 (5.88)	0–21
PHQ-9	12.4 (5.70)	1–27	7.7 (5.79)	0–26
WHODAS Rasch	19.8 (3.90)	10.09–27.90	14.3 (5.74)	0–26.26
RPQ baseline	33.9 (14.41)	0–61	22.9 (14.82)	0–54

*Note*

FAB-TBI = Fear Avoidance Behavior after Traumatic Brain Injury Questionnaire

GAD-7 = General Anxiety Questionnaire 7

PHQ-9 = Patient Health Questionnaire

WHODAS = World Health Organization Disability Assessment

RPQ = Rivermead Postconcussion Symptoms Questionnaire

SD = Standard Deviation

### Associations with FAB-TBI

FAB-TBI scores demonstrate a normal, unimodal distribution both at baseline (Appendix 2a) and following treatment (Appendix 2b). Scores for both FAB-TBI and the single-item fear avoidance measure decreased on average following treatment, as expected, as treatments aimed to reduce fear avoidance behavior (Appendix 1, Appendix 2).

Correlations between the single-item avoidance measure and the individual FAB-TBI items are shown in Table 3. These correlations are fairly uniform within the range of 0.4–0.6, though with some relatively low correlations (< 0.4) with FAB-TBI items loading on the cogniphobia factor.

**Table 3:** Spearman’s correlations between the single-item fear avoidance measure and all items of FAB-TBI, at Baseline and Posttreatment

FAB-TBI Item	Factor*	Correlation (ρ) at baseline	Correlation (ρ) posttreatment
1. I have put parts of my life on hold.	Activity avoidance	0.46	0.51
2. I have avoided my usual activities.	Activity avoidance	0.57	0.54
3. I cannot do activities which (might) make my symptoms worse.	Activity avoidance	0.64	0.67
4. My work might harm my brain.	Activity avoidance/ cogniphobia	0.47	0.27
5. I should not do my normal work with my present symptoms.	Activity avoidance	0.42	0.49
6. My head pain is telling me that I have something dangerously wrong.	Cogniphobia	0.35	0.31
7. I worry that when I have to think or concentrate too hard that I will bring on a headache.	Cogniphobia	0.28	0.37
8. My headaches put my head and brain at risk for the rest of my life.	Cogniphobia	0.37	0.23
9. I purposely avoid doing activities that might elicit a headache.	Cogniphobia	0.59	0.59
10. I’m afraid that I might make my headache pain worse by concentrating too much or being too mentally active.	Cogniphobia	0.39	0.48
11. I wouldn’t have this much pain if there weren’t something potentially dangerous going on in my head.	Cogniphobia	0.41	0.37
12. I avoid external reminders of a stressful experience (for example, people, places, conversations, activities, objects, or situations).	Failed to load onto any factor	0.46	0.40
13. I stop what I am doing when my symptoms start to get worse.	Symptom avoidance	0.45	0.41
14. If I know that something will make my symptoms worse I don’t do it anymore.	Symptom avoidance	0.62	0.61
15. Because of my symptoms most days I spend more time resting than doing activities.	Activity avoidance	0.52	0.53
16. Most days my symptoms keep me from doing much at all.	Activity avoidance	0.59	0.37

*Noteρ* = Spearman’s Rank Correlation Coefficient. Correlations between the single-item fear avoidance measure with every item of the FAB-TBI, at baseline and posttreatment. *Factor structure was originally described by Snell et al. [11]. Factors include activity avoidance, cogniphobia, and symptom avoidance

### Agreement with FAB-TBI

Agreement was assessed between FAB-TBI and the single-item fear avoidance measure using weighted kappa. Moderate agreement was observed between both measures, where baseline measured κ = 0.51 and posttreatment measured κ = 0.45 [21]. The agreement of the change in scores (e.g. posttreatment minus baseline scores) between the single-item fear avoidance measure and FAB-TBI indicated fair agreement, where κ = 0.28. The distribution of differences scores (the inherently ordinal single-item avoidance measure subtracted from discretized (ordinal) FAB-TBI total scores) were approximately normal, suggested no systematic under- or over-estimation (see figures Appendix 3a and 3b in supplemental online material).

### Convergent validity

At baseline, a moderately strong positive correlation was observed between the single-item fear avoidance measure and FAB-TBI (*ρ* = 0.67, *p* < .001) The single-item fear avoidance measure correlated moderately with anxiety (GAD-7; *ρ* = 0.34, *p* < .001), depression (PHQ-9; *ρ* = 0.43, *p* < .001), post-concussion symptoms (RPQ; *ρ =* 0.50, *p* < .001), and disability (WHODAS; *ρ* = 0.43, *p* < .001). The FAB-TBI was more strongly correlated with these measures: anxiety (*ρ* = 0.53; *p* < .001), depression (*ρ* = 0.71; *p* < .001), post-concussion symptoms (*ρ =* 0.73; *p* < .001) and disability (*ρ* = 0.66; *p* < .001).

Posttreatment, there was a moderate correlation between the single-item fear avoidance measure and FAB-TBI (*ρ =* 0.63, *p* < .001). Following treatment, the single-item fear avoidance measure correlated moderately with anxiety (GAD-7; *ρ* = 0.38, *p* < .001), depression (PHQ-9; *ρ* = 0.47, *p* < .001), post-concussion symptoms (RPQ; *ρ* = 0.53, *p* < .001), and disability (WHODAS; *ρ* = 0.53, *p* < .001). Again, the FAB-TBI was more strongly correlated with these measures: anxiety (*ρ* = 0.59; *p* < .001), depression (*ρ* = 0.64; *p* < .001), post-concussion symptoms (*ρ* = 0.72; *p* < .001) and disability (*ρ* = 0.71; *p* < .001). The correlation between the change scores from baseline to posttreatment for the single-item fear avoidance measure and FAB-TBI was moderate (*ρ* = 0.45, *p* < .001).

## Discussion

The present study examined the criterion validity of a single-item fear avoidance measure after mild TBI. We observed a moderately strong positive correlation between the single-item fear avoidance measure and FAB-TBI at baseline, indicating that these two scales may measure the same construct, or at least that there is overlap in what they measure. The strength of these correlations approached the benchmark of > 0.70 suggested by previous studies as indicator of criterion validity of a single-item scale [22–25]. The single-item fear avoidance measure correlated similarly (*ρ* = 0.4–0.6) with the majority of the FAB-TBI items, but somewhat lower on FAB-TBI items that load on cogniphobia, suggesting that it is less able to capture this dimension of the fear avoidance construct. Agreement between (discretized) FAB-TBI and the single-item fear avoidance measure was moderate both at baseline (κ = 0.51) and posttreatment (κ = 0.45), which is greater than chance but less than the ideal threshold of 0.8 [26]. Taken together, these results suggest that the single-item fear avoidance measure has moderate criterion validity, but is not a substitute for the full 16-item FAB-TBI questionnaire. The single-item of fear avoidance demonstrated adequate convergent validity by correlating with measures of anxiety, depression, post-concussion symptoms, and disability. However, correlations between the single-item fear avoidance measure and related constructs were somewhat weaker than the correlations between FAB-TBI and related constructs. The stronger associations may be explained by the fact that FAB-TBI is a multi-item measure of fear avoidance, and is better able to capture a broader range of fear avoidance behavior.

Descriptive analyses suggested an additional limitation of the single-item fear avoidance scale. Terwee et al. [26] recommended that no more than 15% of participants should achieve the highest and lowest scores on a given scale. Having strong floor and ceiling effects can indicate limited content validity and reliability as the measure is not able to accurately distinguish between people who have varying degrees of symptom severity. In the present study, 22% of the sample scored either 1 (lowest possible score) or 10 (highest possible score) on the single-item fear avoidance measure at baseline. While ceiling effects largely decreased posttreatment, which may be partly because of regression to the mean, floor effects notably increased. The single-item fear avoidance measure was sensitive to change from pre- to posttreatment and the magnitude of change was correlated with the change on the FAB-TBI. However, the floor and ceiling effects suggest that the single-item avoidance measure may miss, underestimate, or overestimate changes in people with very high or very low fear avoidance.

The main appeal of single-item scales is their practicality and efficiency. The single-item fear avoidance measure may be a useful screening and monitoring tool for patients with mTBI when constrained for time. The full version of the FAB-TBI could be administered if a patient’s single-item scores are elevated, for instance scores greater than 5. In this study, scores greater than 5 on the single-item fear avoidance measure were associated with FAB-TBI scores indicating increased fear avoidance behavior based on normative data from concussion clinics in Canada [3] (see Appendix Fig. 4). Supplementing a core battery of questionnaires with the single-item fear avoidance measure is more advantageous than not assessing fear avoidance behavior at all. The early assessment of fear avoidance behavior following mTBI can help clinicians identify and target maladaptive coping strategies sooner and improve outcomes [5]. Additionally, a single-item measure is useful for repeated measurements, such as in the case of ecological momentary assessment. Having a brief measure can help monitor the progression of a patient’s fear avoidance behavior over the course of treatment.

In the present study, the sample was predominantly White and female, only included treatment-seeking patients, and excluded older adults. Thus, the findings may not generalize to the broader population. As such, further validation of the single-item avoidance measure is warranted.

## Conclusion

The single-item fear avoidance measure may be a useful screening and monitoring tool for patients with mTBI when constrained for time. However, it is not a substitute for the FAB-TBI questionnaire, which should be used to assess fear avoidance after mild TBI when greater precision is desirable.

## Electronic supplementary material

Below is the link to the electronic supplementary material.

Supplementary Material 1

## Data Availability

No datasets were generated or analysed during the current study.

## Abbreviations

mTBI: mild Traumatic Brain Injury
FAB-TBI: Fear Avoidance Behavior after Traumatic Brain Injury
TBI: Traumatic Brain Injury
PHQ-9: Patient Health Questionnaire 9
GAD-7: General Anxiety Questionnaire 7
WHODAS: World Health Organization Disability Assessment
RPQ: Rivermead Postconcussion Symptoms Questionnaire
IQR: Interquartile Range
LOC: Loss of Consciousness
PTA: Post-Traumatic Amnesia
