# Supplementary material for: Criterion validity of a single-item measure of fear avoidance behavior following mild traumatic brain injury
Source: BMC Neurol. 2024 Sep 28;24:363. doi: 10.1186/s12883-024-03861-3 (PMC11437642; doi:10.1186/s12883-024-03861-3)
Supplement: Supplementary file 1 — Supplementary Material 1 [file 12883_2024_3861_MOESM1_ESM.docx]

**Appendix 1a**

*Distribution of the Single-Item Measure of Fear Avoidance Scores at Baseline*

*Note*. This graph represents the distribution of scores on the single-item measure of fear avoidance at baseline. This measure was a Likert scale, where values range from 1 to 10.

**Appendix 1b**

*Distribution of the Single-Item Measure of Fear Avoidance Scores Posttreatment*

*Note*. This graph represents the distribution of scores on the single-item measure of fear avoidance posttreatment. This measure was a Likert scale, where values range from 1 to 10.

**Appendix 2a**
*Distribution of FAB-TBI Scores at Baseline*

*Note*. The graph above represents the distribution of scores for FAB-TBI at baseline. Scores underwent a Rasch transformation, described in more depth by Snell and colleagues (2020a).

FAB-TBI = Fear Avoidance Behavior after Traumatic Brain Injury Questionnaire

**Appendix 2b**

*Distribution of FAB-TBI Scores Posttreatment*

*Note*. The graph above represents the distribution of scores for FAB-TBI posttreatment. Scores underwent a Rasch transformation, described in more depth by Snell and colleagues (2020a).

FAB-TBI = Fear Avoidance Behavior after Traumatic Brain Injury Questionnaire

**Appendix 3a**

*Distribution of FAB-TBI Scores at Baseline Following Ordinal Transformation*

*Note.* The graph above represents the distribution of scores for discretized FAB-TBI scores at baseline. To discretized the scores (e.g. transform nominal data into ordinal data), we multiplied FAB-TBI Rasch scores by 10/48 and rounded the products to whole numbers.

**Appendix 3b**

*Distribution of FAB-TBI Scores Posttreatment Following Ordinal Transformation*

*Note.* The graph above represents the distribution of scores for discretized FAB-TBI scores posttreatment. To discretized the scores (e.g. transform nominal data into ordinal data), we multiplied FAB-TBI Rasch scores by 10/48 and rounded the products to whole numbers.

**Appendix 4**

*Linear Model of Single-Item and FAB-TBI Scores*

*Note*. The graph above represents the relationship between the single-item fear avoidance measure and FAB-TBI at baseline using a linear model. Single item scores are plotted on the x-axis, while FAB-TBI scores are plotted on the y-axis. Single item scores of 5 correspond to approximately a FAB-TBI Rasch score of 22, which is approximately the 50^th^ percentile of scores based on normative data from concussion clinics in Canada [3].
